# Distinct phospholipase A2 enzymes regulate prostaglandin E2 and F2alpha production by bovine endometrial epithelial cells

**DOI:** 10.1186/1477-7827-5-16

**Published:** 2007-04-25

**Authors:** Patricia K Tithof, Mary P Roberts, Wei Guan, Mona Elgayyar, James D Godkin

**Affiliations:** 1 The University of Tennessee College of Veterinary Medicine, TN, USA; 2 The University of Tennessee, Department of Animal Science, Knoxville, TN, USA

## Abstract

**Background:**

The rate-limiting step in prostaglandin (PG) biosynthesis is catalyzed by phospholipase A2 (PLA2) enzymes which hydrolyze arachidonic acid from membrane phospholipids. Despite their importance in uterine PG production, little is known concerning the specific PLA2 enzymes that regulate arachidonic acid liberation in the uterine endometrium. The objectives of this study were to evaluate the expression and activities of calcium-independent Group VI and Group IVC PLA2 (PLA2G6 and PLA2G4C) and calcium-dependent Group IVA PLA2 (PLA2G4A) enzymes in the regulation of bovine uterine endometrial epithelial cell PG production.

**Methods:**

Bovine endometrial epithelial cells in culture were treated with oxytocin, interferon-tau and the PLA2G6 inhibitor bromoenol lactone, alone and in combination. Concentrations of PGF2alpha and PGE2 released into the medium were analyzed. Western blot analysis was performed on cellular protein to determine the effects of treatments on expression of PLA2G4A, PLA2G6 and PLA2G4C. Group-specific PLA2 activity assays were performed on cell lysates following treatment with oxytocin, interferon-tau or vehicle (control), alone and in combination. To further evaluate the role of specific PLA2 enzymes in uterine cell PG biosynthesis, cells were transfected with cDNAs encoding human PLA2G6 and PLA24C, treated as described above and PG assays performed.

**Results:**

Constitutive cell production of PGF2alpha was about two-fold higher than PGE2. Oxytocin stimulated production of both PGs but the increase of PGF2alpha was significantly greater. Interferon-tau diminished oxytocin stimulation of both PGs. The PLA2G6 inhibitor, bromoenol lactone, abolished oxytocin-stimulated production of PGF2alpha. Treatments had little effect on PLA2G4A protein expression. In contrast, oxytocin enhanced expression of PLA2G6 and this effect was diminished in the presence of interferon-tau. Expression of PLA2G4C was barely detectable in control and oxytocin treated cells but it was enhanced in cells treated with interferon-tau. Oxytocin stimulated PLA2 activity in assays designed to evaluate PLA2G6 activity and interferon-tau inhibited this response. In assays designed to measure PLA2G4C activity, only interferon-tau was stimulatory. Cells overexpressing PLA2G6 produced similar quantities of the two PGs and these values were significantly higher than PG production by non-transfected cells. Oxytocin stimulated production of both PGs and this response was inhibited by interferon-tau. Bromoenol lactone inhibited oxtocin stimulation of PGF2alpha production but stimulated PGE2 production, both in the absence and presence of oxytocin. Cells over-expressing PLA2G4C produced more PGE2 than PGF2alpha and interferon-tau stimulated PGE2 production.

**Conclusion:**

Results from these studies indicate that oxytocin stimulation of uterine PGF2alpha production is mediated, at least in part, by up-regulation of PLA2G6 expression and activity. In addition to its known inhibitory effect on oxytocin receptor expression, interferon-tau represses oxytocin-stimulated PLA2G6 expression and activity and this contributes to diminished PGF2alpha production. Furthermore, endometrial cell PGE2 biosynthesis was associated with PLA2G4C expression and activity and interferon-tau was stimulatory to this process.

## Background

In domestic ruminants (sheep, cattle, goats) estrous cyclicity is regulated by uterine PGF_2α_-induced demise of the corpus luteum [1,2]. PGF_2α _is released from endometrial luminal and superficial glandular epithelium in an episodic fashion toward the end of the estrous cycle. Oxytocin (OT), of neurohypophyseal and luteal origin, is believed to bind endometrial OT receptors and initiate pulsatile PGF_2α _secretion, which in turn, stimulates release of luteal OT and creates a positive feedback loop that results in several series of pulses of short duration which are effective in causing luteolysis [3,4]. During early pregnancy, the conceptus protein interferon tau (IFNT) binds to endometrial receptors and attenuates episodic PGF_2α _secretion, thereby rescuing the CL from regression and maintaining progesterone production [5-7].

Release of arachidonic acid (AA) from membrane glycerophospholipids is catalyzed by phospholipase A_2 _enzymes and is considered the rate-limiting step in PG biosynthesis [8]. Released AA is then metabolized to PGG_2 _by a cyclooxygenase reaction and then to PGH_2 _by a peroxidase reaction, both mediated by cyclooxygenase (COX) -1 and/or -2. Terminal PG synthases, which exhibit tissue-specific distribution, then convert PGH_2 _to bioactive PGs including PGF_2α_, PGE_2_, PGD_2 _and PGI_2_.

Although PLA2 activation catalyzes the rate-limiting step in PG biosynthesis, most investigations of the mechanisms and regulation of uterine PG production have focused on the activation and expression of down-stream enzymes, such as the cyclooxygenases and PG synthases. The few studies [9-12] that have examined the role of PLA2 in uterine PG production in domestic ruminants have focused on a single enzyme, cPLA2α (Group IVA PLA2 or PLA2G4A). Surprisingly, none of these studies identified consistent changes in expression of protein or mRNA for PLA2G4A in association with alterations in PG production. In contrast, non-specific PLA2 inhibitors significantly diminished both basal and agonist-induced PG production [10-12].

Mammalian cells contain structurally diverse PLA2 enzymes that differ in their location and regulation [reviewed in [13] and [14]]. At least 14 groups, and numerous subgroups, of PLA2s have been classified. These groups include four main families of PLA2s, the low molecular weight secretory PLA2s, the cytosolic PLA2G4s, the calcium-independent PLA2G6s and the platelet activating factor (PAF) acid hydrolases (PLA2G7 and PLA2G8). The intracellular PLA2G4 and PLA2G6 enzymes are specifically important in the regulation of PG biosynthesis because their sites of action are the perinuclear membranes where down-stream AA metabolizing enzymes (cyclooxygenases and PG synthases) reside.

The first cytosolic PLA2 discovered was PLA2G4A and it has received, by far, the most attention. It is a 749 amino acid, 85 kDa protein that migrates at 100–112 kDa in polyacrylamide gels. It is present in the cytosol of resting cells and upon activation by a variety of agonists, undergoes Ca^++^-directed translocation to perinuclear membranes. The N-terminal Ca^++^-dependent lipid binding (CaLB) or C2 domain is essential for membrane association and responsible for translocation in response to stimuli that increase intracellular calcium [14+]. Gene disruption studies have indicated an important role for PLA2G4A in normal fertility, eicosanoid production from inflammatory cells, brain injury and allergic response in mice [15-17]. Mice with a null mutation for the gene have fewer implantation sites, smaller litters and fail to undergo labor at term.

Several additional Group IV PLA2 enzymes have been cloned recently, including IVB, IVC, IVD, IVE and IVF [18-20]. Of these enzymes, IVC (PLA2γ) is of interest because it is constitutively associated with membranes, the major site of prostaglandin biosynthesis, lacks the C2 domain and does not require Ca^++ ^for activation. Although first associated with membrane remodeling, recent studies demonstrate that PLA2G4C functions as a signaling PLA2 linked to PGE_2 _production [21].

In the literature, the term calcium-independent PLA2, has generally referred to the Group 6 family of PLA2 enzymes [13], despite the fact that Group 4C is Ca^++^-independent as well. Two distinct subclasses of Group 6 have been identified and characterized, 6A (also referred to as iPLA2 and iPLA2B) and 6B (iPLA2γ). More recently several novel iPLA2s have been identified, including iPLA2δ, iPLA2ε, iPLA2ζ and iPLA2η[22]. These novel iPLA2 family members display primarily lysophospholipase, triacylglycerol lipase and/or transacylase activities rather than PLA2 activity. Group 6A has been cloned and characterized in several species and cell types [23-25]. It has a mass of 85–88 kDa, contains 7–8 terminal ankyrin repeats, and a consensus lipase motif. Several splice variants of the enzyme are expressed yielding multiple molecular weight isoforms [25]. Recently, expression of a 50 kDa Group 6 enzyme in rat myometrium was associated with agonist-induced uterine contractions [26].

All PLA2 enzymes catalyze the hydrolysis of the sn-2 ester bond of phospholipids to produce free fatty acids and lsophospholipid [13,14]. A distinguishing characteristic of PLA2G4A is that it exhibits greater selectivity for lipids containing arachidonic acid than other cytosolic PLA2s. For this reason, PLA2G4A has been the enzyme primarily associated with direct generation of arachidonic acid and initiation of the eicosanoid cascade. However, PLA2G4C [21] and PLA2G6 [32,33] have been associated with agonist-stimulated arachidonic acid release and eicosanoid biosynthesis. In addition, it has been shown that PLA2G6 may mediate release of arachidonic acid directly by hydrolysis of arachidonate-containing plasmalogens or through an indirect, multi-step process involving hydrolysis of diacyl lipids to arachidonyl lysophosphatidylcholine [34].

The objectives of the present study were to investigate the expression and activities of Group 4A, 4C and 6A PLA2 enzymes in association with PGF_2α _and PGE_2 _production in bovine endometrial epithelial cell cultures. Results indicate that in addition to PLA2G4A, PLA2G6 and PLA2G4C contribute to regulation of uterine cell PG production and these latter two enzymes are activated in response to oxytocin and INFT, respectively.

## Methods

### Materials

Bovine endometrial epithelial (BEE) cells were from Cell Applications, Inc. (Catalog no. B932-05, San Diego, CA). Dulbecco minimum essential medium (MEM), Ham F-12, Hank's buffered saline solution (HBSS), gentamicin, oxytocin, PDBu, bovine serum albumin, fetal bovine serum, horse serum, aprotinin, leupeptin and pepstatin were from Sigma Chemical Co (St Louis, MO). Enzyme Immunoassay kits for PGE_2 _(catalog no. 900-001) and PGF_2α _(catalog no. 901-069) were from Assay Designs, Inc (Ann Arbor, MI). 1-palmitoyl-2-(1-^14^C) arachidonyl-phosphotidylcholine (^14^C-AA-PC), 1-palmitoyl-2-(1-^14^C) oleoyl-phosphotidylcholine (^14^C-OA-PC) and 1-palmitoyl-2-(1-^14^C) linoleoyl-phosphotidylcholine (1-^14^C-LA) were from Amersham Corp (Arlington Heights, IL). Group 4A PLA_2 _antibody (SC-454) was from Santa Cruz Biotechnology, Inc (Santa Cruz, CA), Group 6 PLA_2 _antibody (Anti-iPLA_2_, #07-169) was from Upstate Cell Signaling Solutions (Lake Placid, NY) and the anti-Group 4C was a gift from Dr. C.C. Leslie (University of Colorado, Boulder, CO). Recombinant ovine IFNτ (antiviral activity, 1 × 10^8 ^U/mg) was donated kindly by Dr. F.W. Bazer (Texas A&M University, College Station, TX). The PLA2G6 inhibitor, bromoenol lactone (BEL) and the PLA2G4 and PLA2G6 inhibitor methyl arachidonyl fluorophosphonate (MAFP) were from Cayman Chemical (Ann Arbor, MI) and the PLA2G4A inhibitor, pyrrolidine-1 (PYR-1), was a gift from Dr. Michael Gelb (University of Washington, Seattle WA). Tissue culture plasticwares were from Benton Dickinson and Co. (Franklin Lakes, NJ). Unless noted otherwise, additional chemicals were from Sigma Chemical Corp.

### Cell culture

Bovine endometrial epithelial cells were cultured and propagated by procedures suggested by Cell Applications, Inc. According to the distributor, the cells, isolated from epithelium of bovine endometrium and cryopreserved at second passage, can be cultured and propagated for at least 10 doublings. Briefly, cells were seeded in tissue culture dishes of the appropriate size for each experiment (see below) at a concentration of 0.5 × 10^5 ^cells per ml in culture medium (40% Hams F-12, 40% MEM, 200 U insulin/L, 50 μg/ml gentamicin, 10% FBS and 10% horse serum) at 37°C in a humidified atmosphere of 95% air and 5% CO_2 _and culture medium was changed every other day.

### PG assays

Cells were grown to ~90% confluence in 6-well plates, washed three times with HBSS and cultured for 6 h in unsupplemented Hams F-12/MEM with the following treatments: control, oxytocin (OT, 0.1 μM), IFNT (50 ng/ml), the PLA2G6 inhibitor BEL (7.5 μM), IFNT + OT and BEL + OT. Vehicles, Hams F-12/MEM for OT and IFNT, and DMSO for the inhibitor were added to each well. Treatments were performed in triplicate and the experiment was repeated (n = 6). At the end of the incubation period, medium was harvested and stored frozen at -20°C. Prostaglandin F_2α _and E_2 _assays were performed with enzyme immunoassay kits from Assay Designs, Inc., according to supplier's instructions. Inter- and intraassay coefficients of variation (n = 12) were 9% and 11%, respectively.

### Western blot analysis

Western blot analysis was performed as described previously (27). Briefly, cells grown to confluence in 60 mm dishes were treated with vehicle (control), OT and IFNT (50 ng and 1000 ng), alone and in combination for 6 h as described above. Treatments were performed in triplicate. Cells were lysed in RIPA buffer (50 mM Tris-HCl, 150 mM NaCl, 1% SDS, 0.5% sodium deoxycholate, 1 mM DTT, 100 μM PMSF, 1% NP-40, and 1 × protease inhibitor cocktail (Roche Applied Science, Indianapolis, IN), sonicated and centrifuged (10,000 g, 20 minutes). Supernatants from each treatment were combined and 30 μg protein from each treatment was subjected to 10% SDS-polyacrylamide gel electrophoresis and transferred onto nitrocellulose membranes. Non-specific binding sites were blocked with 5% non-fat dry milk and membranes were incubated with antibodies to PLA2G4A, PLA2G4C, and PLA2G6 in 1:800, 1:400 and 1:1000 dilutions, respectively, overnight at 4°C. Membranes were rinsed 3 times and protein bands were visualized using an enhanced fluorescent reagent (ECL Western blotting detection kit, Amersham Biosciences, Piscataway, NJ).

### Over expression of PLA2G4C and PLA2G6A in BEE cells

Full-length cDNAs encoding human PLA2G4C (accession number XM_009119) and PLA2G6A (accession number XM_039248) were generated via PCR using templates from human coronary artery endothelial cells. Primers for PLA2G4C were: upstream 5'CCG CAG TGC ACC ATG GGA AGC TC 3', downstream 5'C TCC CAG CTA CCC GGC CAC GTT C 3'. Primers for PLA2G6A were: upstream 5' G GCG GGC ACC GCC ACT GGA GC 3', downstream 5'GAA TGC CCC ATG CGT CTC CCA G 3'. The cDNAs were ligated into the PCR2.1-TOPO plasmid vector of the TA cloning system (Invitrogen Corp, Carlsbad, CA), subcloned and sequenced. Homologies with the human PLA2G4C and PLA2G6A were >99%. The cDNAs were ligated into the PCR3.1 expression vector (Invitrogen Corp, Carlsbad, CA). Plasmids were incorporated into BEE cells using the Superfect™ (Qiagen Inc, Valencia, CA) transfection system according to manufacturers' instructions. Over expression of PLA2G4C and PLA2G6 was confirmed by Western blot analysis. Cells transfected with PLA2G6 were treated for 6 h with vehicle (control), IFNT (50 ng/ml), OT (0.1 μM), OT and IFNT, BEL (7.5 μM), and BEL and OT. Cells transfected with PLA2G4C were treated for 6 h with vehicle (control), IFNT (50 ng/ml), OT (0.1 μM), OT and IFNT. Treatments were performed in triplicate and repeated (n = 6). Prostaglandin E_2 _and F_2α _assays were performed as described above.

### PLA_2 _activity in cellular homogenates

PLA_2 _assays were performed on cellular lysates, as described previously [27], following treatment of intact cells. Briefly, cells were grown to 80% confluence. Treatments, OT, IFNT, alone and in combination, were applied for 3 h. Some treatments included the inhibitors, BEL, MAFP and PYR-1, which were applied 30 min before other treatments. Concentrations of treatments were as listed above unless otherwise noted. Experiments were performed in triplicate and repeated twice. At the end of the incubation period, cells were washed with Ca^++^-free PBS containing 5 mM EGTA and 1 mM phenylmethyl-sulfonylchloride (PMSF), lysed in homogenizing buffer (50 mM Tris HCl, pH 7.4, 0.5 mM dithiothreitol, 20% glycerol, 1 μg/ml leupeptin, 10 μg/ml aprotinin, 1 mM PMSF and sonicated two times for 10 seconds on ice. The substrates 1-palmitoyl-2-[arachidonoyl] phophatidylcholine (cold AA-PC) and 1-palmitoyl-2-[arachidonoyl-1-^14^C] phosphatidylcholine (^14^C-AA-PC), 1-palmitoyl-2-[linoleoyl] phosphatidylcholine (cold LA-PC), 1-palmitoyl-2-[linoleoyl-1-^14^C] phosphatidlycholine (^14^C-LA-PC), 1-palmitoyl-2-[oleoyl] phosphatidylcholine (OA-PC) and 1-palmitoyl-2-[oleoyl-1-^14^C] phosphatidylcholine (^14^C-OA-PC) were used to assay for PLA_2 _activity. The substrates were dried under nitrogen and resuspended by sonication in assay buffer (10 mM Hepes, pH 7.5) to a final optimum concentration of cold to radiolabeled substrate as determined previously. In preliminary studies, calcium-dependent and – independent activities were identified. Calcium-dependent activity (primarily PLA2G4A) assays were performed with AA-PC and ^14^C-AA-PC in the presence of 5 mM CaCl_2 _and calcium-independent activity (primarily PLA2G6 and PLA2G4C) assays were performed using the same substrates but in the absence of CaCl_2 _and the presence of EGTA (5 mM). Assays optimized for identification of PLA2G4C utilized OA-PC and ^14^C-OA-PC in the absence of calcium (this enzyme is arachidonoyl-nonselective and Ca^++^-independent) and assays optimized for PLA2G6 utilized LA-PC and ^14^C-LA-PC in the absence of Ca^++ ^and presence of 400 μM Triton-X and 0.8 mM ATP which enhance the activity of this enzyme [28]. Experiments were terminated by addition of chloroform-methanol, 2:1 (v/v), the chloroform layer was extracted and lipids separated by thin-layer chromatography (hexane: diethyl ether: glacial acetic acid, 7:3:0.2). Lipids were visualized with I_2 _vapor, the zones corresponding to fatty acid and phospholipids were excised and radioactivity determined by scintillation counting.

### Statistical analysis

Data for PG production and PLA2 activity are presented as means +/- SEM and subjected to least squares analysis of variance using the general linear models procedure of the Statistical Analysis System (SAS Institute, Cary, NC). Sources of variance included experiments, treatments and their interactions. Individual comparisons of means were made using Student-Neuman-Keuls test in which the independent variables were the treatments and the dependent variables were the levels of PG or PLA2 activity produced. Differences were considered statistically significant when P < 0.05.

## Results

### Prostaglandin production

**Figure 1:**
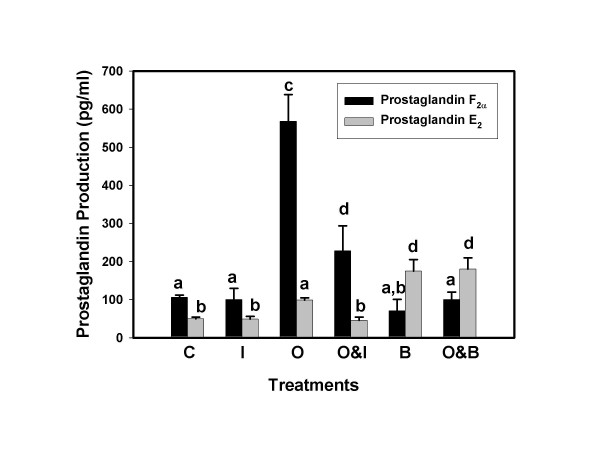
**Prostaglandin F_2α _and E_2 _production by BEE cells**. Cell culture medium was harvested 6 h after addition of treatments and concentrations of PGs were analyzed by ELISA. Treatments included, Control, C; IFNT, I; Oxytocin, O; Oxytocin and IFNT, O&I, Bromoenol lactone, B; and Oxytocin and Bromoenol lactone, O&B. Treatments were performed in triplicate and repeated (n = 6). Data are expressed as means and SEM. Columns with different superscripts are significantly different (p < 0.05).

The effects of treatment on PGF_2α _and PGE_2 _production by bovine endometrial epithelial cells are illustrated in Figure 1. In the absence of stimulation (control), cells produced about twice the amount of PGF_2α _as PGE_2 _(p < 0.05). Oxytocin stimulated PGF_2α _and PGE_2 _production about five fold and two fold, respectively. Alone, IFNT had little effect on the production of either PG but it significantly inhibited the stimulatory effect of oxytocin on PGF_2α _production and numerically diminished PGE_2 _production. Alone, BEL, the PLA2G6 inhibitor [30], had little effect on PGF_2α _production but enhanced PGE_2 _synthesis. In combination with oxytocin, BEL completely abolished the stimulatory effect of oxytocin on PGF_2α _but not PGE_2 _production.

**Figure 2:**
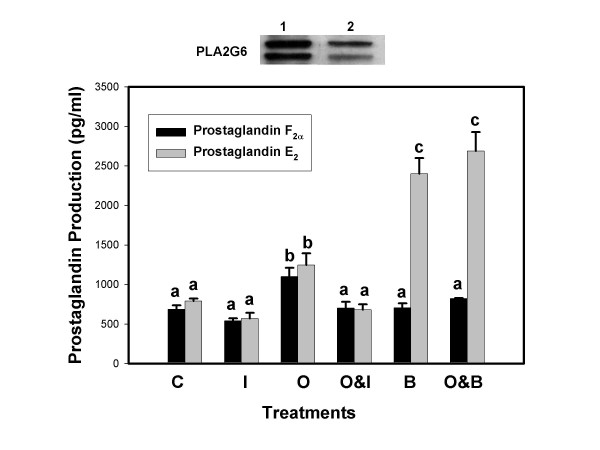
**Prostaglandin F_2α _and E_2 _production by BEE cells over-expressing PLA2G6**. Culture medium from BEE cells, transfected with PLA2G6 cDNA, was harvested 6 h after exposure to treatments and concentrations of PGs were analyzed by ELISA. Treatments included Control, C; IFNT, I; Oxytocin, O; :Oxytocin and IFNT, O&I, Bromoenol lactone, B; Oxytocin and Bromoenol lactone, B&O. Treatments were performed in triplicate and repeated (n = 6). Data are expressed as means and SEM. Columns with different superscripts are significantly different (p < 0.05). Western blot at top of figure compares PLA2G6 expression in transfected cells (lane 1) and non-transfected cells (lane 2).

Prostaglandin production by BEE cells transfected with PLA2G6 plasmids, is illustrated in Figure 2. Overexpression (OE) of PLA2G6 in BEE cells significantly increased constitutive production of PGF_2α _and PGE_2 _compared to non-transfected cells (compare values in Fig. 2 to Fig. 1). Oxytocin significantly increased production of both PGs by about 50% compared to non-stimulated OE (control) cells. Alone, IFNT had little effect on PG production but it diminished, significantly, the stimulatory effect of oxytocin when the treatments were combined. The PLA2G6 inhibitor BEL, alone, did not affect PGF_2α _production compared to controls, but it abolished oxytocin stimulation of PGF_2α _production when the two treatments were combined. Conversely, BEL dramatically enhanced PGE_2 _production, alone and in combination with oxytocin.

**Figure 3:**
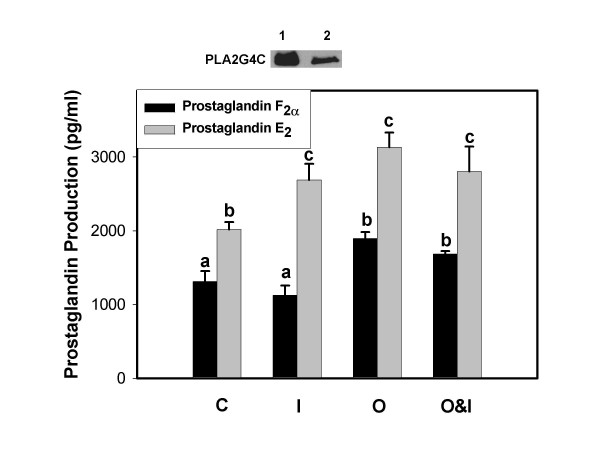
**Prostaglandin F_2α _and E_2 _production by BEE cells over-expressing PLA2G4C**. Culture medium from BEE cells, transfected with PLA2G4C cDNA, was harvested 6 h after exposure to treatments and concentrations of PGs were analyzed by ELISA. Treatments included Control, C; IFNT, I; Oxytocin, O, Oxytocin and IFNT, O&I. Data are expressed as means and SEM. Treatments were performed in triplicate and repeated (n = 6). Columns with different superscripts are significantly different (p < 0.05). Western blot at top of figure compares PLA2G4C expression in transfected cells (lane 1) and non-transfected cells (lane 2).

Figure 3 illustrates results of prostaglandin production by BEE cells overexpressing the PLA2G4C enzyme. Production of both PGs was significantly enhanced compared to non-transfected cells and the increase in PGE_2 _was significantly greater than that of PGF_2α_. Alone, IFNT had little effect on PGF_2α _production but significantly stimulated PGE_2 _production. Oxytocin stimulated production of both PGs compared to control PLA2G4C transfected cells and the effect on PGE_2 _production was greater. When combined, IFNT diminished oxytocin stimulation of PGF_2α _but PGE_2 _production values were similar to oxytocin and IFNT treatments, alone.

### PLA_2 _protein expression

**Figure 4:**
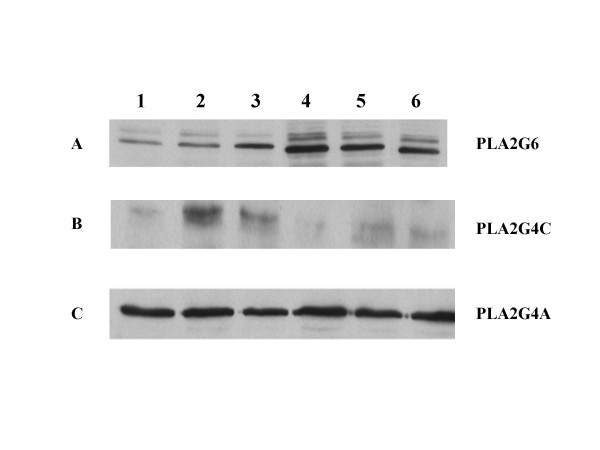
**Oxytocin and interferon-tau differentially regulate expression of PLA2 enzymes**. BEE cells were treated for 6 h with vehicle (control, lane 1), 50 ng IFNT (lane 2), 1000 ng IFNT (lane 3), oxytocin (lane 4), oxytocin plus 50 ng IFNT (lane 5), and oxytocin plus 1000 ng IFNT (lane 6). Western blot analyses were performed on cellular proteins using antibodies against **A**, PLA2G6; **B**, PLA2G4C and **C**, PLA2G4A as described in the Methods.

Western blot analysis of cellular proteins from BEE cells demonstrated that the PLA2G4A antibody recognized a protein which migrated at ~110 kD, the PLA2G6 antibody recognized protein which migrated at 85 kD and the PLA2G4C antibody cross-reacted with a protein migrating at 61 kD (Figure 4). Two isoforms of PLA2G6 were identified, a major band at 85 kd, and a minor band of slightly greater mass. Similar result have been observed previously and likely the result of alternative splicing of the PLA2G6 transcript resulting in multiple isoforms of the enzyme [25]. Cells were treated for 6 h with vehicle (control, lane 1), IFNT (50 ng, lane 2), IFNT(1000 ng, lane 3), oxytocin(0.1 μM, lane 4), oxytocin (0.1 μM) plus 50 ng IFNT (lane 5) and oxytocin (0.1 μM) plus 1000 ng IFNT (lane 6) to determine the effects of these treatments on PLA_2 _isotype expression. As shown in Figure 4A, expression of PLA2G6 (Fig 4A) was influenced by treatments. Oxytocin up-regulated PLA2G6 (Fig 4A, lane 4) and this effect was diminished by IFNT treatments (Fig 4A, lanes 5 and 6). Expression of PLA2G4C (Fig 4B) was barely detectable, except when cells were treated with IFNT, which enhanced expression of the enzyme (Fig 4B, lanes 2, 3, 5 and 6). In contrast, PLA2G4A was expressed at similar levels in all treatment groups (Figure 4C).

### PLA_2 _activity assays

**Figure 5:**
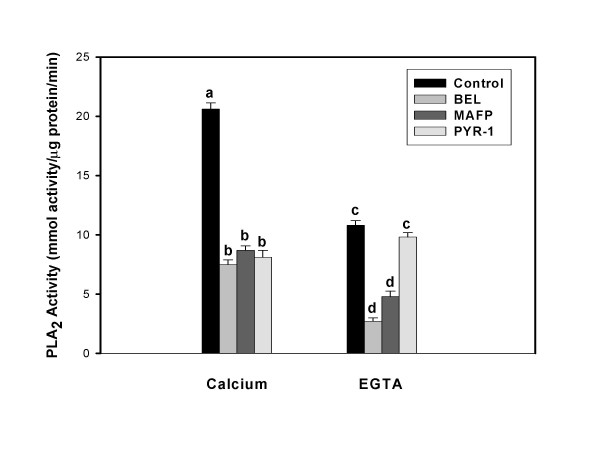
**BEE cells exhibit both Ca^++^-dependent and Ca^++^-independent PLA2 activities**. PLA2 activity assays were performed on cellular lysates following treatment of intact cells, as described in the Methods. Ca^++^-dependent PLA2 activity assays were performed with AA-PC and ^14^C-AA-PC in the presence of 5 mM CaCl_2_and Ca^++^-independent assays were performed using the same substrates but in the absence of CaCL_2 _and the presence of 5 mM EGTA. Treatments included the PLA2G6 inhibitor BEL, the PLA2G4 and PLA2G6 inhibitor MAFP and the PLA2G4A inhibitor PYR-1. Data from columns with different superscripts are significantly different (p < 0.05).

Cell lysate PLA_2 _assays were performed in an attempt to identify enzyme-specific activities based on differences in substrate selectivity, Ca^++ ^requirements, cofactor-dependent activity amplification and specific inhibitor effects. Figure 5 illustrates that BEE cells exhibit both Ca^++^-dependent activity (primarily PLA2G4A) and Ca^++^-independent activity (primarily PLA2G6 and PLA2G4C). Calcium-dependent activity was inhibited by PYR-1, a PLA2G4A-specific inhibitor [31], MAFP, a G4 and G6 inhibitor [30] and BEL (a PLA2G6-specific inhibitor) due to the fact that both G4C and G6 PLA_2 _enzymes do not require Ca^++ ^for activity but function in its presence. Calcium-independent activity was inhibited by BEL and MAFP, but not PYR-1, which inhibits only the Ca^++^-dependent PLA2G4A.

**Figure 6:**
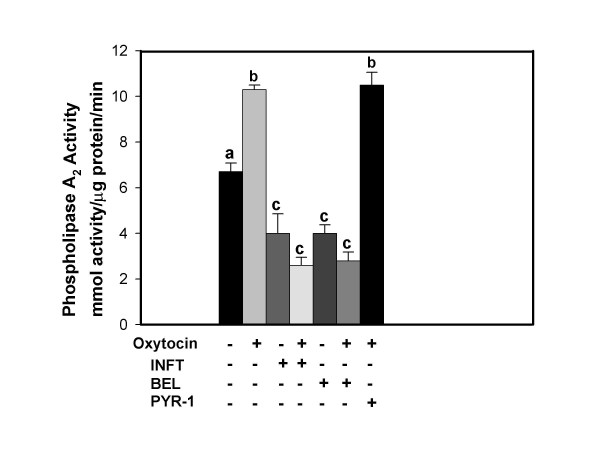
**Oxytocin stimulated PLA2G6 activity is blocked by IFNT**. BEE cells were treated for 3 h with vehicle control, oxytocin, IFNT, BEL or PYR-1, alone or in combination. The symbols, + and -, indicate the presence or absence of treatments, respectively. PLA2G6 activity assays were performed on cellular lysates as described in the Methods. Inhibition of activity by BEL but not PYR-1 demonstrates that PLA2G6 activity was measured. Columns with different superscripts are significantly different (p < 0.05).

Figure 6 depicts the results of PLA_2 _assays designed specifically to evaluate PLA2G6 activity in homogenates of BEE cells following treated of intact cells for 3 h with or without oxytocin in the presence and absence of IFNT, BEL or PYR-1. Oxytocin significantly stimulated PLA2G6 activity and IFNT inhibited this response. Inhibition of oxytocin stimulation of activity by BEL but not PYR-1 confirms that PLA2G6 activity was measured.

**Figure 7:**
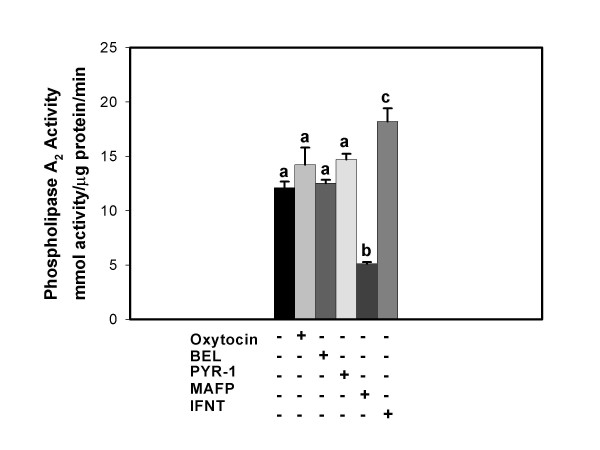
**PLA2G4C activity is enhanced by IFNT but not oxytocin**. BEE cells were treated for 3 h with vehicle control, oxytocin, BEL, PYR-1, MAFP or IFNT. The symbols, + and -, indicate the presence or absence of treatments, respectively. PLA2G4C activity assays were performed on cellular lysates as described in the Methods. Inhibition of activity by MAFP but not PYR-1 or BEL demonstrates that PLA2G4C activity was measured. Columns with different superscripts are significantly different (p < 0.05).

Results from PLA_2 _assays designed to measure PLA2G4C activity are illustrated in Figure 7. BEE cells were treated for 3 h with vehicle (control), oxytocin, BEL, PYR-1, MAFP or IFNT. Considerable constitutive activity was present and this activity was inhibited by the G4 and G6 PLA2 inhibitor MAFP but not the G6-specific inhibitor BEL or the G4A-specific inhibitor PYR-1. Group IVC PLA2 activity was significantly stimulated by IFNT.

## Discussion

Prostaglandins are essential regulators of numerous reproductive processes but, despite their primary involvement in fertility regulation, the cellular and molecular mechanisms that regulate prostaglandin production are poorly understood. The rate-limiting step in prostanoid biosynthesis is at the level of PLA2 enzymes but, surprisingly, little is known concerning the regulatory function of specific PLA2 isoforms that mediate arachidonic acid release and are likely coupled to downstream enzymes that synthesize the different PGs in reproductive tissues. Although in vivo studies may provide the most complete physiological conditions in which to evaluate uterine biology, they are not best suited for analysis of some cellular mechanisms involving biochemical pathways. For this reason, we turned to in vitro cell cultures to evaluate PLA2 regulation of PG biosynthesis. To our knowledge, this is the first study to demonstrate that expression and activity of the calcium-independent PLA2G6 and PLA2G4C in endometrial epithelial cells are differentially regulated by oxytocin and IFNT, resulting in altered prostaglandin biosynthesis.

Three lines of evidence support the concept that PLA2G6 is involved in oxytocin stimulation of endometrial PGF_2α _production. One, the PLA2G6 inhibitor BEL abolished oxytocin stimulation of PGF_2α _production. Two, oxytocin increased expression of PLA2G6 as determined by Western blot analysis; and three, PLA2G6-specific activity was increased by oxytocin and this response was blocked by BEL. Similarly, the results indicate that IFNT diminished oxytocin stimulated PLA2G6 expression (Figure 4A, Western blot analysis) and activity (Figure 6) resulting in diminished PGF_2α _production (Figure 1). These results do not exclude PLA2G4A from playing a role in oxytocin-stimulated uterine PG production. Indeed, PLA2G4A knock-out mice fail to initiate labor at term. On the other hand, the results presented may help to explain why previous studies [10-12] failed to identify consistent changes in PLA2G4A expression or activity in response to oxytocin.

The observation that BEL inhibition of PLA2G6 activity blocked oxytocin stimulation of PGF_2α _production led to studies that examined the effects of PLA2G6 overexpression on PG production. Cells that over expressed this enzyme produced significantly more PGF_2α _and PGE_2 _than control non-transfected cells. Oxytocin increased production of both PGs and IFNT diminished these responses. As expected, BEL inhibited oxytocin-stimulated PGF_2α _production. In contrast, BEL, alone or in combination with oxytocin, greatly enhanced PGE_2 _production. Increased PGE_2 _production in response to BEL was also observed in non-transfected BEE cells and, in previous studies [29], we have repeatedly observed stimulation of PGE_2 _production by BEL in primary cultures of ovine endometrial epithelial cells. Although BEL is a relatively selective PLA2 inhibitor at the concentration used, it has also been shown to inhibit Mg^++^-dependent phosphatidic acid phosphohydrolase (PAP-1), an enzyme that catalyzes the dephosphorylation of phosphatidic acid to diacylglycerol [30]. Change in PAP-1 activity would not explain the observed increase in PGE_2_. Alteration of the PGE_2 _: PGF_2α _ratios could be affected at the level of the PG synthases. To our knowledge, there is no evidence that BEL affects PGE synthase expression or activity. In addition, studies in human monocytes [32] and rat mesangial cells [33] have demonstrated that stimulation of the arachadonic acid/PGE cascade is mediated by PLA2G6 activation and BEL inhibited agonist-stimulated PGE_2 _production in these cells. These data would argue against a stimulatory activity of BEL on PGE_2 _synthase.

Functional coupling between different PLA2 isoforms, COX-2 and specific PG synthases has been suggested as a regulatory mechanism of prostanoid biosynthesis in a variety of cell types [36-38]. Individual isoforms, coupled to specific signaling stimuli, may act on different cellular pools of arachidonic acid at different locations in membranes. Inhibition of PLA2G6 with BEL blocked oxytocin stimulated PGF_2α _production but stimulated PGE_2 _production. One possible explanation for the differential effects of BEL on PG production may be that PLA2G6 is functionally coupled to PGF_2α _production which is stimulated by oxytocin and inhibition of PLA2G6 blocks this response. Conversely, inhibition of PLA2G6 may be permissive to activation of other PLA2 isoforms, such as PLA2G4C, which may be functionally coupled to PGE_2 _production. Clearly, confirmation of this hypothesis will require additional studies.

During early pregnancy in ruminants, IFNT not only suppresses episodic release of PGF_2α _from the endometrium, but it also enhances production of PGE_2 _[39-41]. PGE_2 _has been proposed to have multiple roles in the maintenance of early pregnancy, including action as a luteotrophic or luteoprotective signal and as a mediator of endometrial receptivity [38,39]. The mechanism by which IFNT up-regulates PGE_2 _production is unknown but Arosh et al. [39] have shown that it does not enhance PGE_2 _synthase expression in bovine endometrium. We propose IFNT regulation of PGE_2 _production involves, at least in part, modulation of expression and activity of a PLA2 isoform. Results from cell-free PLA_2 _assays and Western blot analysis demonstrated that PLA2G4C activity and expression were up-regulated by IFNT. Over expression of PLA2G4C in BEE cells increased production of both PGs but the increased PGE_2 _was significantly greater than the increased PGF_2α_. Treatment of these cells with IFNT significantly stimulated PGE_2 _production. Together, these results indicate that the PLA2G4C enzyme is coupled to PGE_2 _production and this activity is positively regulated by IFNT.

Luteolysis, resulting from episodic secretion of PGF_2α_, is acknowledged, generally, to be orchestrated progesterone, estradiol and oxytocin acting through their respective endometrial receptors [1,2]. During early pregnancy, IFNT diminishes expression of the estrogen receptor- alpha and the oxytocin receptor thereby blocking the OT-PGF_2α _feedback loop and preventing luteolysis [41,42]. There can be little doubt that diminished oxytocin receptor expression contributes to early pregnancy maintenance in ruminants, but some studies have indicated that IFNt-mediated repression of oxytocin receptors is not absolute. For example, Burgess et al. [43] demonstrated that PGFM release, following bolus inter-arterial oxytocin injection, was similar in day 14–16 cyclic and pregnant ewes, indicating that some oxytocin receptor activity remains during early pregnancy; and Mann et al. [44] demonstrated in cattle that oxytocin receptor concentrations were not a limiting factor in oxytocin stimulated PGFM release. Based on the results from the present study, it is suggested that in addition to repressing oxytocin receptor expression, IFNT diminishes oxytocin stimulation of PLA2G6-linked PGF_2α _production and enhances PLA2G4C-linked PGE_2 _production.

## Conclusion

Together, the data indicate that uterine PG production is regulated, at least in part, by the expression and activity of PLA2 enzymes and that specific PLA2 isotypes may regulate the production of specific PGs.

## Competing interests

The author(s) declare that they have no competing interests.

## Authors' contributions

PKT designed and supervised completion of the PLA2 assays, Western blot analyses, over-expression studies and contributed to the drafting of the manuscript. MPR performed the PG assays. WG performed the over-expression studies and PLA_2 _assays. ME perform the Western blot analyses. JDG designed the study, overall, and drafted the manuscript. All authors read and approved the final version of the manuscript.
